# Targeting CD47 for cancer immunotherapy

**DOI:** 10.1186/s13045-021-01197-w

**Published:** 2021-10-30

**Authors:** Zhongxing Jiang, Hao Sun, Jifeng Yu, Wenzhi Tian, Yongping Song

**Affiliations:** 1 Department of Hematology, the First Affiliated Hospital of Zhengzhou University, Zhengzhou, 450052 Henan China; 2 Department of Radiation Therapy, the First Affiliated Hospital of Zhengzhou University, Zhengzhou, 450052 Henan China; 3 Henan International Joint Laboratory of Nuclear Protein Gene Regulation, Henan University College of Medicine, Kaifeng, 475004 Henan China; 4 ImmuneOnco Biopharmaceuticals (Shanghai) Co., Ltd., Shanghai, 201203 China; 5 Department of Hematology, the Affiliated Cancer Hospital of Zhengzhou University and Henan Cancer Hospital, Zhengzhou, 450008 Henan China

**Keywords:** CD47, Signal regulatory protein α (SIRPα), Monoclonal antibody (mAbs), Bi-specific antibody (BsAbs), Fusion proteins, Cancer immunotherapy, Clinical trials

## Abstract

Much progress has been made in targeting CD47 for cancer immunotherapy in solid tumors (ST) and hematological malignancies. We summarized the CD47-related clinical research and analyzed the research trend both in the USA and in China. As of August 28, 2021, there are a total 23 related therapeutic agents with 46 clinical trials in the NCT registry platform. Among these trials, 29 are in ST, 14 in hematological malignancies and 3 in both solid tumor and hematological malignancy. The ST include gastric cancer, head and neck squamous cell carcinoma and leiomyosarcoma, while the hematological malignancies include non-Hodgkin's lymphoma, acute myeloid leukemia, myelodysplastic syndrome, multiple myeloma and chronic myeloid leukemia. Majority of the CD47-related clinical trials are at the early phases, such as 31 at phase I, 14 at phase II and 1 at phase III in the USA and 9, 6, 1, in China, respectively. The targets and spectrums of mechanism of action include 26 with mono-specific and 20 with bi-specific targets in the USA and 13 with mono-specific and 3 with bi-specific targets in China. The new generation CD47 antibodies have demonstrated promising results, and it is highly hopeful that some candidate agents will emerge and make into clinical application to meet the urgent needs of patients.

## Introduction

Cluster of differentiation 47 (CD47) is a trans-membrane protein ubiquitously expressed on human cells but overexpressed on many types of tumor cells. It is a cell surface glycoprotein molecule, belonging to the immunoglobulin superfamily, binding to various proteins including integrin, thrombospondin-1 and signal regulatory protein α (SIRPα). CD47 is an important tumor antigen for the development and progression of various cancers. The interaction of CD47 with SIRPα triggers a "don't eat me" signal to the macrophages, inhibiting phagocytosis. Thus, overexpression of CD47 enables tumor cells to evade immune surveillance via the blockade of phagocytic mechanisms. CD47 blockade alone is not sufficient to trigger macrophage anti-tumor activity. Macrophages also need an “eat-me” (pro-phagocytic) signal. In recent years, accumulating data suggest that the CD47-SIRPα axis is a key immune checkpoint in different cancers including hematological malignancies, similar to that of the PD-1/PD-L1 checkpoint for solid tumors. CD47-SIRPα blockade has emerged as a next-generation immune checkpoint disruption strategy in various malignancies after PD-1/PD-L1. Many CD47 monoclonal antibodies (mAbs) not only block CD47 from engaging SIRPα, but also engage the activating Fc gamma receptor (FcγR) on macrophages. Together they deliver a potent phagocytic signal to macrophages.

In addition to the CD47-SIRPα signaling pathway, significance of CD47 expression has been investigated in different malignancies. Association between CD47 expression, clinical characteristics and prognosis in patients with different malignancies, including advanced non-small cell lung cancer [1], gastric cancer [2], colorectal adenocarcinoma [3] and pancreatic neuroendocrine tumor [4] has been explored. For example, in patients with high-grade lung neuroendocrine tumors, the high expression of CD47 was strongly associated with a worse progression-free survival, especially in patients with a Ki-67 > 40% [1]. CD47 expression also has correlation with adverse clinicopathologic features and an unfavorable prognosis in colorectal adenocarcinoma [3]. Similarly, CD47 has been found playing important roles in hematological malignancies, including in non-Hodgkin lymphomas (NHL), lymphoblastic lymphoma/acute lymphoblastic leukemia (LBL/ALL), acute myeloid leukemia (AML) and multiple myeloma (MM) [5, 6]. CD47 has become a potential therapeutic target and is being studied in various preclinical studies and clinical trials to prove its safety and effectiveness in the treatment of hematological neoplasms [7], and continues to grow rapidly around the world [7–10]. Many therapeutic products targeting CD47 are being developed, including anti-CD47 mAbs, bi-specific antibodies (BsAbs) to CD47 and other molecules, as well as SIRPα-related fusion proteins. Some of the therapeutic products have demonstrated promising results in clinical research. This review will help readers to comprehensively understand the advances of clinical development of CD47-targeted therapeutic products including mAbs, SIRPα-Fc fusion proteins and BsAbs. The trend of technological innovation development is also discussed.

## The revival story of CD47 clinical development

Prior to becoming the current new focus in the field, the prospect of CD47 just a few years ago was quite dim. In 2017, a phase I clinical trial of CD47 mAb Ti-061 in Europe (EudraCT number, 2016-004372-22) was terminated [11]. Then, in 2018, CD47 mAb CC-90002 failed in the phase I clinical trial (NCT02641002). The severe hemolytic reaction caused by hemagglutination (HA) induced by CD47 monoclonal antibody was the main problem of those clinical failures. The repetitive setbacks made the prospect of developing CD47 for cancer treatment very pessimistic. In 2019, combination of CD47 mAb magrolimab and azacitidine in the treatment of acute myeloid leukemia (AML)/myelodysplastic syndrome (MDS) showed excellent and sustainable efficacy with manageable hematological toxicity when the initial dose of 1 mg/kg was given one week before the treatment dose (10–30 mg/kg) [12]. With that positive result, since then, CD47-targeted drug development gained renewed interests, was resurrected and thrusted into a new era.

## SIRPα-CD47 signal pathway

### SIRPα

**Fig. 1:**
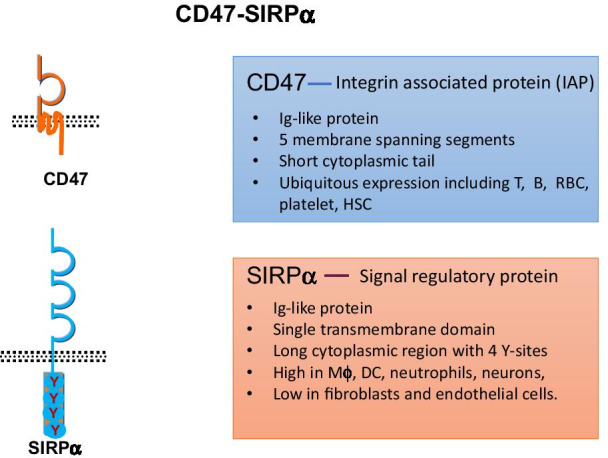
CD47-SIRPα

SIRPα consists of three immunoglobulin-like extracellular domains and putative tyrosine phosphorylation sites in the cytoplasmic region (Fig. 1) [13]. Various growth factors such as integrin can mediate cell adhesion and induce the tyrosine phosphorylation of SIRPα [13, 14]. The tyrosine-phosphorylated sites of SIRPα bind to the protein tyrosine phosphatases SHP-1 and SHP-2 through its cytoplasmic region at the cell membrane in response to extracellular stimuli [15]. SIRPα is abundantly expressed on neurons, macrophages, dendritic cells and neutrophils [16, 17]. However, SIRPα is barely detectable in red blood cells (RBCs) or in T or B lymphocytes, whereas CD47 is ubiquitously expressed on a variety of human cells including RBCs and platelets, and the expression of different isoforms of CD47 protein is tissue specific [18–20].

### CD47

CD47 is a ligand for the extracellular region of SIRPα [21]. The extracellular region of CD47 is associated with the integrin β3 subunit (Fig. 1) [22]. Most CD47-mediated cellular responses involve the activation of integrins, in particular, αvβ3 or αIIbβ3 [18]. The N-terminal IgV domain of SIRPα binds to the extracellular Ig domain of CD47 [23, 24]. In contrast to the restricted expression of SIRPα, CD47 is expressed on both healthy and cancer cells. The interaction of SIRPα with CD47 is important for the regulation of migration and phagocytosis and plays a pivotal role in this balance by delivering a "don't eat me signal" [19]. CD47-SIRPα axis has been considered as a promising therapeutic target in different diseases and stem cell therapies [19].

### Mechanism of actions in the CD47-SIRPα signaling pathway

**Fig. 2:**
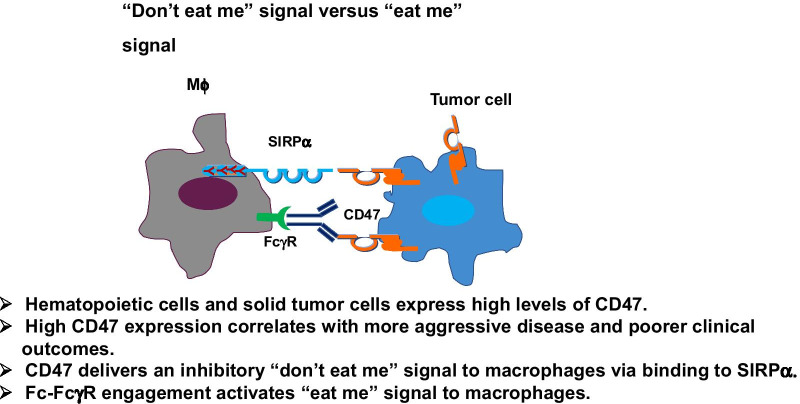
“Don’t eat me” signal versus “eat me” signal

CD47-SIRPα interaction regulates the outcome of myeloid cell-target cell signal transduction (Fig. 2). Signaling through the CD47-SIRPα axis can influence erythrocyte, platelet and hematopoietic stem cell maintenance [25–27]. The expression of CD47 on cancer cells can inhibit myeloid cell-mediated clearance in a manner similar to the inhibition of T cell activity by tumors via PD-1/PD-L1 interaction [28]. Numerous studies had been carried out on the main mechanism of action (MOA) of CD47/SIRPα blockades, by targeting either CD47 or SIRPα.

**Fig. 3:**
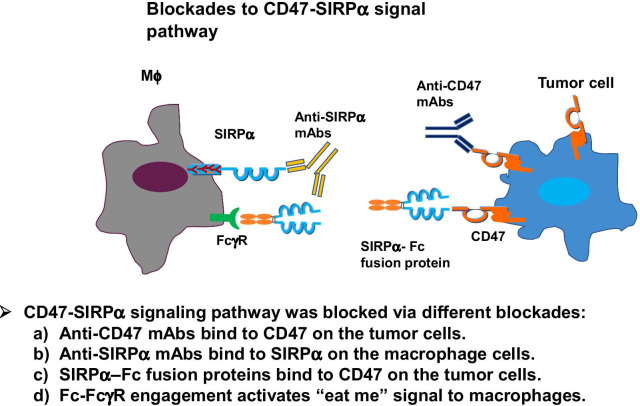
Blockades to CD47-SIRPα signal pathway

Several molecules blocking the CD47-SIRPα axis are currently being developed in the pre-clinical stage (Fig. 3) [19]. Studies demonstrated the potential value of targeting this axis in stem cell transplantation and possibly in diseases such as atherosclerosis and fibrosis [29, 30]. It has been demonstrated that anti-SIRP mAbs SIRP-1 and SIRP-2 disrupt SIRPα/CD47 interaction by two distinct mechanisms: SIRP-1 directly blocks SIRPα/CD47 interaction and induces internalization of SIRPα/Ab complexes that reduce macrophage SIRPα surface levels, whereas SIRP-2 acts via disruption of higher-order SIRPα structures on macrophages. Both SIRP-1 and SIRP-2 engage FcγRII, which is required for single-agent phagocytic activity. Anti-SIRPα antibodies can enhance phagocytosis when combined with tumor-opsonizing anti-CD47 mAbs [8].

In recent years, pre-clinical studies on the concept of tumor microenvironment (TME) have revealed a variety of immune cells interacting with each other and, respectively, regulating tumorigenesis or immune regulation. Research results showed that CD47-SIRPα immune checkpoint pathway played important roles in tumor immune evasion and innate immunotherapy [31]. The expression of CD47 also has an important impact on the construction of TME [32], whereas CD47 deficiency led to cancer stem cell depletion. Hypoxia-inducible factor 1 regulates CD47 expression in breast cancer cells to promote evasion of phagocytosis and maintenance of cancer stem cells [33]. Elevated CD47 expression on some cancer cells protects tumors from innate immune surveillance and limits adaptive antitumor immunity via inhibitory SIRPα signaling in antigen-presenting cells. CD47 also mediates inhibitory thrombospondin-1 signaling in vascular cells, T cells and NK cells; blocking inhibitory CD47 signaling on cytotoxic T cells directly increases tumor cell killing [34].

Pre-clinical results showed that CD47 was more abundantly expressed on AML self-renewing leukemia stem cells (LSC) than their normal counterparts. Increased CD47 expression predicted worse overall survival (OS) in adult AML patients. Furthermore, CD47 mAbs preferentially enabled phagocytosis of AML LSC and inhibited their engraftment in vivo. CD47 mAbs depleted AML and targeted AML LSC in xenograft mouse models [35]. In addition, phagocytosis checkpoints were impaired in MDS. High expression of CD47 on CD34^+^CD38-cells indicated poor clinical prognosis in MDS [36]

Among the anti-CD47 mAbs, many have the differentiated characteristics of binding to CD47 with high affinity, and minimal binding to human erythrocytes, which reduces the potential adverse effect of anemia caused by HA. A potential explanation for the differential binding of CD47 mAbs to different cells could be due to the fact that CD47 associates with different membrane proteins (in *cis*) on different cells. Therefore, certain epitopes may be masked by CD47 binding partners in different cells (such as erythrocyte membrane protein 4.2 and/or Rh complex components [37]), or dependent on CD47 interacting proteins (such as integrin [31]), or physically associated with other membrane proteins (such as VEGFR2) [31]. Because CD47 can be heavily glycosylated with five potential NXT/S sequences in its extracellular IgV domain [38] and/or modified by addition of glycosaminoglycans, differing patterns or extents of carbohydrate additions in different cell types could also explain the differential binding of CD47 antibodies [39]. A further explanation for the differential binding of CD47 mAbs to cancer versus normal cells lies in potential differences in surface mobility of CD47 on different cell types [40] or different densities or distribution of CD47 in the lipid rafts [41]. Furthermore, the binding of CD47 mAbs to CD32a on macrophage can play dual roles: inducing FcγR-mediated phagocytosis of cancer cells and as a scaffold introducing CD47-mediated death signals into tumor cells [42].

It is important to point out that targeting CD47 on tumors can immensely enhance anti-tumor effect of other therapeutic strategies. In addition to the combination of anti-CD47 with regular chemotherapy, it can also be used with biologics, such as combining CD47 blockade with trastuzumab, which eliminates HER2-positive breast cancer cells and overcomes trastuzumab tolerance [43]. Combination of anti-CD47 and other immune checkpoint pathways such as PD-1/PD-L1 had been studied by different research groups [44]. Anticancer bispecific antibody studies focusing on clinical research have been a hotspot recently with many BsAbs developed for clinical applications. In terms of the MOA, six categories of BsAbs had been developed including T-cell redirection, dual checkpoint blockades, dual signaling inhibitions, co-localized blockages, biparatopic BsAbs and tumor-targeted immunomodulators [45]. BsAbs aiming to both CD47 and another target such as CD19 [46], PD-1 (HX009) had been developed with promising results. These mechanisms further expand the potential of anti-CD47 therapy in its clinical application.

## The clinical development of CD47 mAbs in the USA

CD47-related clinical research and trial information was retrieved through PubMed database and the online open resources for clinical trial registration platforms from the US national clinical trials registry (NCT) system www.clinicaltrials.gov and China drug trials registry (CDT) system www.chinadrugtrials.org.cn. Frontiers of CD47 clinical research are summarized, and its clinical trials-related information including organizations, identity numbers, phases, participating centers, conditions, status, enrollment, targets, spectrum of MOA, start date is collected. Clinical trials are summarized based on the territory of pharmaceutical companies: international or China-initiated or—involved as presented in Tables 1 and 2.

**Table 1:** CD47-related clinical trials registered in US national clinical trials registry (NCT) system www.clinicaltrials.gov

Agent	CD47 Isotype	Organization	Mechanism of action	NCT Number	Status	Conditions	Phase	Enrollment	Start dates
AK117	IgG4	Akesobio	A new generation of humanized anti-human CD47 IgG4 mAb,	NCT04349969	Not yet recruiting	Neoplasms Malignant	Phase 1	159	25-Apr-20
				NCT04728334	Recruiting	Neoplasms Malignant	Phase 1	162	27-Jan-21
				NCT04900350	Recruiting	MDS	Phase 1/2	190	18-Jun-21
				NCT04980885	Recruiting	AML	Phase 1/2	160	Jul-21
AO-176	IgG2	Arch Oncology	Fully humanized anti-human CD47 monoclonal antibody	NCT03834948	Recruiting	Solid Tumor	Phase 1/2	183	4-Feb-19
				NCT04445701	Recruiting	MM	Phase 1/2	102	30-Nov-20
CC-90002	IgG4	Celgene	First generation anti-human CD47 antibody	NCT02367196	Completed	Hematological Malignancies	Phase 1	60	12-Mar-15
CPO107 JMT601 (CPO107)	IgG1 (CD20)	Conjupro Biotherapeutics, Inc	Bi-specific SIRP α and anti-CD20 fusion protein. Through interference of CD47/SIRP α interaction to enhance antibody-dependent phagocytosis (ADCP)	NCT04853329	Not yet recruiting	NHL	Phase 1/2	75	15-Aug-21
DSP107	IgG4	KAHR Medical	Bi-functional CD47 and 41BB fusion protein	NCT04440735	Recruiting	Advanced Solid Tumor Non-Small Cell Lung Cancer	Phase 1 Phase 2	100	7-Oct-20
				NCT04937166	Recruiting	AML/MDS CML	Phase 1 Phase 2	36	Sep-21
Evorpacept (ALX148)	IgG1	ALX Oncology	Fusion protein of SIRPα D1 domain and an inactive human IgG1 Fc	NCT03013218	Active, not recruiting	Solid Tumor NHL	Phase 1	174	3-Feb-17
				NCT04417517	Recruiting	MDS	Phase 1	173	2-Oct-20
				NCT04675294	Recruiting	Head and Neck Squamous Cell Carcinoma	Phase 2	111	April 2, 2021
				NCT04675333	Recruiting	Head and Neck Squamous Cell Carcinoma	Phase 2	112	10-May-21
				NCT04755244	Recruiting	AML	Phase 1/2	97	5-May-21
				NCT05002127	Not yet recruiting	Gastroesophageal Junction Adenocarcinoma Gastric Adenocarcinoma	Phase 2/3	450	Aug-21
Hu5F9-G4 Magrolimab(5F9)	IgG4	Gilead Sciences/Forty Seven	Anti-human CD47 mAb	NCT02216409	Completed	Solid Tumor	Phase 1	88	Aug-14
				NCT02678338	Completed	AML/MDS	Phase 1	20	Nov-15
				NCT03248479	Recruiting	Hematological Malignancies	Phase 1	287	8-Sep-17
				NCT03527147	Completed	NHL DLBCL NHL Diffuse Large B Cell Lymphoma	Phase 1	30	19-Jun-18
				NCT04599634	Not yet recruiting	Follicular Lymphoma Marginal Zone Lymphoma Mantle Cell Lymphoma Chronic Lymphocytic Lymphoma B-Cell Lymphoma	Phase 1	76	1-Sep-21
HX009	IgG4	Hanxbio	PD-1/CD47 bi-specific mAb	NCT04097769	Active, not recruiting	Advanced Solid Tumor	Phase 1	20	18-Mar-21
				NCT04886271	Recruiting	Solid Tumor	Phase 2	210	10-May-21
IBI188	IgG4	Innovent Bio	Recombinant human anti-CD47 mAb	NCT03717103	Active, not recruiting	Advanced Malignancies	Phase 1	49	10-Jan-19
				NCT03763149	Active, not recruiting	Advanced Malignancies	Phase 1	42	11-Jan-19
				NCT04485052	Recruiting	AML	Phase 1 Phase 2	126	Apr-21
				NCT04485065	Recruiting	MDS	Phase 1	12	30-Sep-20
				NCT04861948	Recruiting	Solid Tumors Lung Adenocarcinoma Osteosarcoma	Phase 1	120	25-May-21
IBI322	IgG4	Innovent Bio	Bi-specific antibody that can target CD47 and PD-L1	NCT04328831	Recruiting	Advanced Malignancies	Phase 1	218	31-Jul-20
				NCT04338659	Recruiting	Advanced Malignancies	Phase 1	45	Apr-21
				NCT04795128	Recruiting	Hematological Malignancies	Phase 1	230	7-May-21
				NCT04912466	Recruiting	Solid Tumor	Phase 1	36	20-Jun-21
IMC-002	IgG4	ImmuneOncia Therapeutics 3D Medicines	Anti-human CD47 monoclonal antibody	NCT04306224	Recruiting	Solid Tumor NHL	Phase 1	24	5-Jun-20
IMM0306	IgG1	ImmneOnco	Bi-specific antibody targeting CD47 and CD20 at the same time	NCT04746131	Not yet recruiting	NHL	Phase 1	90	15-Aug-21
OSE-172	IgG4	OSE Immunotherapeutics	Humanized IgG4 mAb against SIRPα	NCT04806035	Recruiting	CLL SLL Richter's Transformation Indolent Lymphoma Follicular Lymphoma Marginal Zone Lymphoma Aggressive Lymphoma DLBCL Mediastinal Large B-cell Lymphoma MCL	Phase 1	60	23-Apr-21
PF-07257876	IgG4	Pfizer	Bi-specific antibody against CD47 and PD-L1,	NCT04881045	Not yet recruiting	Non-Small Cell Lung Cancer Head and Neck Squamous Cell Carcinoma Ovarian Cancer	Phase 1	90	27-Aug-21
SHR-1603	IgG4	Jiangsu HengRui Medicine	Humanized IgG4 mAb targeting CD47	NCT03722186	Suspended	Physiological Effects of Drugs Neoplasms by Histologic Type NHL	Phase 1	128	13-Nov-18
SHR2150	IgG4 (TLR7)	HengRui Pharma	Selective TLR7 receptor agonist	NCT04588324	Recruiting	Solid Tumor	Phase 1/2	50	10-Oct-20
SRF231	IgG4	Surface Oncology	Anti-human CD47 mAb	NCT03512340	Completed	Advanced Solid Cancers Hematological Malignancies	Phase 1	148	13-Mar-18
STI-6643	IgG4	Sorrento Therapeutics, Inc	Fully humanized anti-CD47 mAb	NCT04900519	Not yet recruiting	Solid Tumor	Phase 1	24	Aug-21
TG-1801	IgG4	Novimmune SA TG Therapeutics	Bi-specific mAb targeting both CD19 and CD47	NCT03804996	Recruiting	NHL	Phase 1	16	5-Mar-19
TJ011133	IgG4	I-MAB Biopharma	Highly differentiated CD47 monoclonal antibody	NCT04895410	Not yet recruiting	MM	Phase 1	163	13-Jul-21
				NCT04912063	Recruiting	AML/MDS	Phase 1	120	15-Jun-21
TTI-621	IgG1	Trillium Therapeutics Inc	SIRPα-IgG1 Fc, checkpoint inhibitor CD47 blocker,	NCT02663518	Recruiting	Hematological Malignancies Solid Tumor	Phase 1	260	Jan-16
				NCT04996004	Recruiting	Leiomyosarcoma	Phase 1/2	80	25-Jun-21
ZL-1201	IgG4	Zai Lab	Anti-human CD47 mAb	NCT04257617	Recruiting	Advanced Malignancies	Phase 1	66	11-May-20

**Table 2:** CD47-related clinical trials registered in the China drug trials registry (CDT) system www.chinadrugtrials.org.cn

Agent	CD47 Isotype	Organization	Mechanism of action	CDT Number	Status	Conditions	Phase	Enrollment	Start date
IMM0306	IgG1	ImmuneOnco	Bi-specific mAb-Trap CD47 and CD20	CTR20192612	Recruiting	R/R NHL, CD20 +	Phase 1	42	23-Mar-20
HX009	IgG4	Hanxbio	Bi-specific mAb CD47 and PD-1	CTR20211292	Recruiting	Advanced solid tumor	Phase 2	210	12-Jul-21
SHR-1603	IgG4	Jiangsu HengRui Medicine	CD47 mAb	CTR20181964	Suspended	Advanced Solid Tumors	Phase 1	128	5-Nov-18
TJ011133	IgG4	I-MAB Biopharma	CD47 mAb	CTR20210313	Recruiting	R/R advanced solid tumors, NHL	Phase 1	50	18-Mar-21
				CTR20192522	Recruiting	R/R AML, MDS	Phase 1/2	42	19-Dec-19
				CTR20210555	Recruiting	AML, MDS	Phase 1/2	60	29-Mar-21
ZL-1201	IgG4	Zai Lab	CD47 mAb	CTR20210973	Recruiting	Advanced solid tumor	Phase 1	N/A	7-May-21
AK117	IgG4	Akesobio	CD47 mAb	CTR20202684	Recruiting	Advanced solid tumors, NHL	Phase 1	162	29-Dec-20
				CTR20210825	Recruiting	MDS	Phase 1/2	190	12-May-21
				CTR20211305	Active, not Recruiting	AML	Phase 1/2	160	4-Jun-21
IBI188	IgG4	Innovent Bio	CD47 mAb	CTR20210761	Recruiting	Advanced solid tumor	Phase 1	120	12-Apr-21
				CTR20182140	Completed	Advanced Solid Tumors	Phase 1	49	22-Nov-18
				CTR20200938	Recruiting	AML	Phase 1/2	222	30-Jul-20
				CTR20201039	Recruiting	MDS	Phase 1/3	338	23-Jul-20
IMM01	N/A	ImmuneOnco	SIRPα Fc fusion protein targeting CD47	CTR20191531	Recruiting	R/R NHL	Phase 1		20-Aug-19

In the past few years, the clinical research on CD47 mAbs has made rapid progress in the USA. As of August 28, 2021, 46 clinical trials on CD47-targeted therapy were registered on clinicaltrials.gov platform. Different types of cancer patients were studied in these clinical trials, including 29 trials in solid tumor (ST) including gastric cancer (GC), head and neck squamous cell carcinoma (HNSC), and leiomyosarcoma (LMS), 14 trials in hematological malignancies including non-Hodgkin's lymphoma (NHL), AML, MDS, MM and chronic myeloid leukemia (CML), as well as 3 trials in both solid tumors and hematological malignancies. Among these clinical trials, different anti-CD47 mAbs were investigated with various strategies [47]. The types of mAbs and its MOAs were quite diverse (Table 1, 2).

### CC-90002 and TI-061

CC-90002 and TI-061 were the first generation of humanized anti-CD47 antibodies entered into clinical research. The first clinical trial of CC-90002 (NCT02641002) was terminated in late 2018 due to its preliminary mono-therapy data in R/R AML and high-risk MDS that failed to provide sufficient evidence for further dose escalation/expansion [48]. After modification of the drug administration strategy and other procedures, CC-90002 was restarted in the clinical trial (NCT02367196).

CC-90002 is unique among previously reported anti-CD47 bivalent antibodies that it does not promote HA while maintaining high-affinity binding to CD47 and inhibition of the CD47-SIRPα interaction. Studies in a panel of hematological cancer cell lines showed concentration-dependent CC-90002-mediated phagocytosis in lenalidomide-resistant MM cell lines and AML cells from patients. In vivo studies with MM cell line-derived xenograft models established in immunodeficient mice demonstrated significant dose-dependent antitumor activity of CC-90002. Treatment with CC-90002 significantly prolonged survival in an HL-60-disseminated AML model. Mechanistic studies confirmed the binding of CC-90002 to tumor cells and concomitant recruitment of F4-80-positive macrophages into the tumor tissue and an increase in expression of selective chemokines and cytokines of murine origin. Furthermore, the role of macrophages in the CC-90002-mediated antitumor activity was demonstrated by transient depletion of macrophages with liposome-clodronate treatment. In non-human primates, CC-90002 displayed acceptable pharmacokinetic properties and a favorable toxicity profile. These data demonstrate the potential activity of CC-90002 across hematological malignancies and provided basis for clinical studies CC-90002-ST-001 (NCT02367196) and CC-90002-AML-001 (NCT02641002) [49].

### Hu5F9-G4 (5F9)

Hu5F9-G4 (5F9, Magrolimab) is a humanized IgG4 antibody that targets CD47 with high affinity [9, 50]. 5F9 antibody humanization was carried out by grafting its complementarity-determining regions (CDRs) onto a human IgG4 format. This humanized antibody bound monomeric human CD47 with an 8 nM affinity. 5F9 induced potent macrophage-mediated phagocytosis of primary human AML cells in vitro and completely eradicated human AML in vivo, leading to long-term disease-free survival of patient-derived xenografts. Moreover, 5F9 synergized with rituximab to eliminate NHL engraftment and cure xenografted mice [50]. 5F9 has been entered into clinical trials in patients with AML and solid tumors (NCT02216409) [9, 50].

The potential adverse events using anti-CD47 antibodies as cancer therapeutics include anemia and thrombocytopenia. Study showed that Hu47F9-G4 alone or in combination with other antibodies may cause accidental killing of normal hematopoietic cells [51]. For the safety concern, 5F9 used an improved administration scheme in the clinical design [10]. The starting dose of 1 mg/kg was used to remove the aging red blood cells and stimulate the maturation and differentiation of reticulocytes to produce new RBCs. These new RBCs are resistant to destruction at a higher maintenance dose of 30 mg/kg, so it can reduce the side effects of anemia. In terms of treatment, 5F9 adopts four schemes: single drug, combined with chemotherapy, combined with tumor-targeted drugs and combined with immunotherapy. Among them, the single drug approach is mainly for AML and some solid tumors, the combination with azacitidine for the first-line treatment of AML and MDS, the combination with therapeutic antibody for recurrent or refractory non-Hodgkin's lymphoma (R/R NHL) and the combination with immunotherapy PD-1/PD-L1 (avelumab) for ovarian cancer [12].

Relatively low response rates were demonstrated in patients with relapsed/refractory AML/MDS and solid tumors when 5F9 was used as monotherapy [50]. This probably was attributed to the reduced affinity of IgG4 for Fcγ receptor on macrophages, hereby limiting antibody-dependent cellular phagocytosis (ADCP) [52]. Furthermore, plasma samples from patients treated with anti-CD47 mAbs (Hu5F9-G4) showed strong reaction with all RBCs and platelets. This reactivity could be removed by multiple alloabsorptions with papain-treated cells or pooled platelets and/or the use of monoclonal Gamma-clone anti-IgG in the indirect antiglobulin testing [53]. Another phase Ib/II trial in 75 patients with R/R NHL with a combination of 5F9 plus rituximab demonstrated an objective response rate (ORR) of 49% with a complete response (CR) rate of 21% [54]. In another phase Ib study, a combination with azacitidine, a DNA demethylating agent demonstrated a 100% ORR and a 55% CR in eleven patients with untreated MDS and 64% CR in 14 patients with AML previously untreated, respectively [12].

### TTI-621 and TTI-622

TTI-621 is a SIRPα fusion protein targeting CD47 and can block CD47 by using decoy receptor (SIRPα-Fc). As a dual-functioning soluble decoy receptor with the CD47-binding domain of SIRPα fused to an IgG1 Fc region, TTI-621 had been applied intravenously for hematological malignancies and intratumorally for solid tumors and mycosis fungoides [53]. The IgG1 Fc tail of TTI-621 plays a critical role in its antitumor activity, presumably by engaging activating Fcγ receptors on macrophages [52]. TTI-621 exhibits minimal binding to human erythrocytes, which reduces the potential for anemia, thereby differentiating it from other CD47 blocking antibodies. Although 2 patients experienced transaminitis and thrombocytopenia, it is unclear whether the thrombocytopenia was dose-limiting due to limited information on the phase I trial [55]. In cutaneous T-cell lymphoma, Sézary syndrome, peripheral T-cell lymphoma and diffuse large B-cell lymphoma (DLBCL), 0.2 mg/kg TTI-621 produced ORR ranging from 17 to 25%, and several CRs were documented [56]. TTI-621 not only blocks CD47 from engaging SIRPα, but also engages the activating receptor FcγR on macrophages. Together they deliver a potent phagocytic signal to macrophages [55, 57].

TTI-621 clinical trial in relapsed, refractory hematological malignancies showed that systemic administration of TTI-621 could lead to CD47 blockade and dose-dependent increase in cytokines associated with phagocytosis, temporally associated with reversible thrombocytopenia, suggesting enhanced macrophage-mediated clearance of circulating platelets followed by a robust marrow regenerative response [58].

Phase I study of TTI-621 combined with rituximab in 164 patients with B-cell non-Hodgkin lymphoma (B-NHL) or with nivolumab in patients with Hodgkin lymphoma showed TTI-621 was well-tolerated and demonstrated promising therapeutic activity as monotherapy in patients with R/R B-NHL and T-cell non-Hodgkin lymphoma (T-NHL) and combined with rituximab in patients with R/R B-NHL. The ORR was 13% for all patients, 29% (2/7) for diffuse large B-cell lymphoma (DLBCL) and 25% (8/32) for T-cell NHL (T-NHL) with TTI-621 monotherapy and was 21% (5/24) for DLBCL with TTI-621 plus rituximab [55].

Meanwhile, phase I dose escalation study of another CD47-blocker TTI-622 in patients with advanced R/R lymphoma demonstrated 1 patient with stage 4 non-germinal center B-cell (non-GCB) DLBCL (5 prior therapies) initially achieved PR by week 8 and CR by week 36. Preliminary data indicated a dose-dependent increase in PK exposure and target engagement with 1 DLBCL patient having achieved a durable, ongoing CR [57].

The difference between the two drugs lies in the different Fc subtypes. TTI-621 and TTI-622 use IgG1 Fc and IgG4 Fc, respectively. Since the interaction between IgG4 Fc region of TTI-622 and Fc receptor is more limited than IgG1, it is speculated that TTI-622 will transmit a more moderate "phagocytosis" signal to macrophages [55, 57].

### ALX-148

ALX148 is a decoy receptor fusion protein comprised of a SIRPα domain mutated for high affinity CD47 binding and an inactive Fc region for the mitigation of HA and anemia. In the presence of an inert Fc region, the decoy is rendered inactive as monotherapy, which necessitates development in combination with other therapies. Results to date indicate ALX148 is generally well tolerated with moderate adverse events in combination with other anticancer drugs, such as Herceptin and Keytruda for patients with solid tumors [59, 60]. While no complete or partial responses were observed with monotherapy, combination with trastuzumab resulted in a 22% partial response rate in patients with HER2-positive gastric cancers and a 16% partial response rate in combination with pembrolizumab in patients with HNSC. No responses were reported in combination with pembrolizumab in eighteen patients with non-small cell lung cancer [61].

Notably, the molecular weight of ALX148 is only half of the antibody IgG, which can achieve linear pharmacokinetics at about half the dose of anti-CD47 antibody and achieve the occupancy of CD47 target. ALX148 stimulates antitumor properties of innate immune cells by promoting dendritic cell activation, macrophage phagocytosis and a shift of tumor-associated macrophages toward an inflammatory phenotype, whereas it also stimulated the antitumor properties of adaptive immune cells, causing increased T cell effector function, pro-inflammatory cytokine production and a reduction in the number of suppressive cells within the TME [60].

A phase I study of ALX148 in combination with standard anticancer antibodies and chemotherapy regimens in patients with advanced malignancy demonstrated excellent tolerability. Clinical activity in patients with advanced HNSC and GC compares favorably with historic controls (NCT03013218) [62]. Recent clinical data indicated that combination use of ALX148 with standard dosing regimens of trastuzumab, ramucirumab and paclitaxel demonstrated a promising initial confirmed objective response rate of 72% and estimated 12 months OS of 76%. No maximum tolerated dose reached [63].

### AO-176

AO-176 is a fully humanized mAb against CD47. With the IgG2 subtype, it has highly differentiated characteristics and improved safety and effectiveness. Specifically, AO-176 can not only preferentially bind to tumor cells rather than normal cells, more effectively bind tumors in its acidic microenvironment (low pH), but also directly kill tumor cells in a cell autonomous manner, not antibody-dependent cellular cytotoxicity (ADCC) [64]. AO-176 showed negligible binding to RBC and did not induce HA and transient anemia, comparing to other CD47 blocking antibodies. It was found to be well tolerated and devoid of hematological effects in cynomolgus monkeys following repeated dose administrations. [41].

A first-in-human study in patients with advanced solid tumors demonstrated that AO-176 was a well-tolerated, differentiated anti-CD47 therapeutic. Durable anti-tumor activity was observed. Currently, clinical trials of AO-176 combined with paclitaxel in patients with select solid tumors (NCT03834948) and as a single-agent in patients with multiple myeloma (NCT04445701) are ongoing. Preliminary data showed that among the 27 patients enrolled with a median of 4 prior therapies for metastatic disease, one patient with endometrial carcinoma who had not responded to any of 4 prior systemic regimens had a confirmed PR and remained on study for > 1 year. Seven patients had stable disease (SD) as best response, with 2 patients (endometrial carcinoma, gastric cancer) on study for > 6 months [65].

### SRF231

SRF231 is a fully human IgG4 anti-CD47 antibody, which was previously granted orphan drug qualification by FDA for the treatment of patients with multiple myeloma. SRF231 exerts dual antitumor activity in vitro through phagocytosis and cell death with the engagement of activating receptor CD32a. Dependent on activation FcγR, the binding of SRF231 to CD32a on macrophage has a dual role: inducing FcγR-mediated phagocytosis of cancer cells and used as a scaffold to drive CD47-mediated death signals into tumor cells. Preclinical study showed that SRF231 can bind with high affinity to CD47 and kill cancer cells in vitro with a strong antitumor activity. Robust antitumor activity occurred across multiple hematological xenograft models either as a single agent or in combination with rituximab. In tumor-bearing mice, SRF231 increased tumor macrophage infiltration and induction of the macrophage cytokines, mouse chemoattractant protein 1 and macrophage inflammatory protein 1 alpha. Macrophage depletion resulted in diminished SRF231 antitumor activity, underscoring a mechanistic role for macrophage engagement by SRF23. A potential safety advantage of SRF231 is that it does not cause detectable RBC agglutination or phagocytosis [42].

### TG-1801

TG-1801 is a bi-specific mAb targeting both CD19 and CD47. By co-targeting CD47 and CD19, TG-1801 has the potential to overcome the limitations of existing CD47-targeted therapies by avoiding the side effects caused by indiscriminate blockade of CD47 on healthy cells [66]. Currently, there are 2 phase I clinical trials ongoing with TG-1801, NCT03804996 for patients with B-cell lymphoma and NCT04806035 for patients with CLL, SLL, Richter's transformation, indolent lymphoma, follicular lymphoma, marginal zone lymphoma, aggressive lymphoma, DLBCL, mediastinal large B-cell lymphoma, MCL.

### BI 765063 (OSE-172)

BI 765063 (OSE-172) is a humanized IgG4 mAb antagonist of SIRPα, which blocks the “don't eat me” signal of the SIRPα/CD47 axis. BI 765063 binds to the V1 SIRPα allele with high affinity and to the V2 SIRPα allele with low affinity. BI 765063 lacks SIRPγ binding to preserve T-cell activation. BI 765063 monotherapy dose escalation phase I study in 50 patients with advanced solid tumors including ovarian (9), colorectal (8), lung (5), breast (4), melanoma (3) and kidney (3) demonstrated well-tolerated safety, pharmacokinetics and efficacy [67]. One patient with hepatocellular carcinoma (HCC) with liver and lung metastases and 7 prior lines of therapy showed a durable partial response maintained for 27 weeks on treatment (ongoing). There was an increase in CD8 T-cell infiltration and activation on treatment. An increase in PD-L1 expression on tumor cells 2 weeks after first dosing was also observed (NCT03990233, NCT04653142) [67].

### Other candidates

With more than 30 registered clinical trials ongoing in the NCT system alone, there are a few more CD47 mono-specific or bi-specific mAbs or fusion proteins, such as DSP107 (NCT04937166, NCT04440735) [68], IMC-002 (NCT04306224) [69] and STI-6643 (NCT04900519) that are joining. More clinical trials are under way, and it is highly hopeful that in the near future some of these therapeutics will emerge as the stars and enter into clinical application to meet the urgent needs of patients.

## The clinical development of anti-CD47 mAbs in China

In recent years, much progress has also been made in anti-CD47 therapeutic development in China. According to the information from the national pharmaceutical products administration (NMPA) website, at least 17 CD47-targeted drugs have been approved by NMPA for clinical research, accounting for more than half of the CD47-targeted agents globally. Currently, there are 15 registered clinical trials using 8 different therapeutic products in the CDT system; 9 of them are in patients with solid tumors and 6 are in patients with AML/MDS. The eight therapeutic agents from China include 6 mAbs, 2 BsAbs and 1 fusion protein, and 7 of them are registered in both NCT and CDT registry platforms, one drug is registered in CDT platform only. Here we summarize and review the advances on these potential therapeutic products from China.

### TJC4 (TJ011133, Lemzoparlimab)

TJC4 (also known as TJ011133, lemzoparlimab) is a highly differentiated RBC-sparing CD47 antibody with a unique epitope. It maintains strong antitumor activity and minimizes the binding with normal RBCs, so as to reduce the HA possibly caused by some CD47 mAbs. Preclinical results showed that monotherapy with lemzoparlimab inhibited tumor growth completely and extended the OS of treated mice in a patient-derived AML xenograft model. TJC4 barely affected the hemoglobin and other blood indexes in the tested animals, showing significantly better safety than 5F9. Compared to the CD47 mAb 5F9, TJC4 binds more weakly to human erythrocytes and does not cause HA [70]. Initial results of lemzoparlimab (NCT04202003) in phase I/IIa study indicated a well-tolerated safety profile with certain clinical efficacy among 5 R/R AML and MDS patients with 2–4 prior therapies. Of special note, one patient with primary refractory AML achieved morphologic leukemia-free state (MLFS) after 2 cycles of lemzoparlimab treatment at 1 mg/kg [70].

The preliminary results of phase I clinical trial in combination with azacitidine in untreated AML or MDS (NCT04202003) have provided further clinical validation in cancer patient therapy. Currently, there are 2 ongoing phase I clinical trials in the NCT systems, NCT04895410 in MM and NCT04912063 in AML/MDS, respectively (Table 1).

### IBI188 and IBI322

IBI188 is a recombinant human anti-CD47 mAb that specifically blocks the CD47-SIRPα axis. In vivo and in vitro experiments showed that IBI188 could bind to CD47 antigen on the surface of tumor cells and block CD47-SIRPα signal pathway, inhibit the "don't eat me" signal transmitted by CD47, so as to enable macrophages to recognize tumor cells and enable the anti-tumor effect. Preclinical results showed that IBI188 treatments up-regulate cell movement and inflammation-related genes in macrophages. Synergism was observed when combined with an anti-CD20 therapeutic antibody, whose function depends on antibody-dependent cellular cytotoxicity/phagocytosis (ADCC/ADCP). Enhanced anti-tumor efficacy from IBI188 was observed in AML xenograft models. Notably, IBI188 treatment increased vascular endothelial growth factor-A (VEGF-A) levels in a solid tumor model, and combined treatment with an anti-VEGF-A antibody and IBI188 resulted in an enhanced anti-tumor effect [71].

Currently, there are 5 ongoing IBI188 clinical trials in the NCT system NCT04861948, NCT04485065, NCT04485052, NCT03717103, NCT03763149 targeting different malignancies (Table 1). IBI188 has been approved for clinical trials both in China and the USA.

IBI322 is a newer BsAb that targets CD47 and PD-L1 at the same time. It has been approved for clinical research in China in January 2020. Its indications are for solid tumors and blood tumors. The preliminary pharmacodynamics study on dose escalation PET imaging with 89Zr-labeled antibodies was promising for PK/PD modeling and safety prediction [72]. A recent study demonstrated that IBI322 selectively bound to CD47^+^PD-L1^+^ tumor cells, effectively inhibited CD47-SIRPα signal and triggered strong tumor cell phagocytosis in vitro, but only with minimal impact on CD47 single positive cells such as human RBCs. In addition, as a dual blocker of innate and adaptive immune checkpoints, IBI322 effectively accumulated in PD-L1-positive tumors and demonstrated synergistic activity in inducing complete tumor regression in vivo. Furthermore, IBI322 showed only marginal RBCs depletion and was well tolerated in non-human primates after repeated weekly injections [73]

Currently, 4 IBI322-related clinical trials have been registered in the NCT system, NCT04912466, NCT04338659, NCT04328831 and NCT04795128, for different malignancies (Table 1).

### HX009

HX009 is a novel humanized antibody against CD47 and PD-1 simultaneously. Theoretically, Hx009 can inhibit tumor immune escape and release immunosuppression of immune checkpoints by simultaneously activating innate and acquired immune responses, so as to achieve the synergistic anti-tumor effect. In a preclinical study, HX009 significantly inhibited tumor growth in mouse xenograft models. A phase I clinical trial (NCT04097769) evaluated the safety and efficacy of HX009 in 21 patients with advanced malignancies, including 7 patients with colorectal cancer, 3 with squamous cell carcinoma, 2 with endometrial cancer, 3 with breast cancer, 1 with malignant epithelioid mesothelioma, 1 with gallbladder cancer, 1 with pancreatic cancer, 1 with glioblastoma, 1 with ovarian cancer and 1 with gastroesophageal junction adenocarcinoma. Patients had received a median of 3 (range 1–9) prior anti-cancer regimens. Among the 18 patients who have had at least one post-baseline tumor assessments, 3 patients achieved PR and 6 patients with best overall response of stable disease [74]. At present, there are 2 ongoing clinical trials in the NCT system, NCT04097769, NCT04886271. Clinical research on HX009 has been conducted simultaneously in Australia and China (Table 1).

### IMM01, IMM0306, IMM2902

IMM01 is a new generation of SIRPα Fc fusion protein targeting CD47. Preclinical experimental studies have proved that IMM01 has a dual mechanism of blocking CD47- SIRPα signaling pathway, as well as activating the "Eat me" signal via Fc-FcγR engagement (Unpublished data, personal communication). Aiming at the immune regulatory CD47, IMM01 can activate macrophages to phagocytose tumor cells and to present tumor antigens to T cells. Thus, it executes strong antitumor activity in vivo as monotherapy. Currently, there is 1 ongoing clinical trial in the NCT system (CTR20191531) (Table 1). Preliminary results showed that some patients had unprecedented response, especially for patients with R/R classical Hodgkin's lymphoma (cHL). Among the five enrolled subjects with cHL, one patient with PD-1 drug-resistant disease achieved PR for 57 weeks and still continues to receive treatment, and 3 patients achieved SD, with an overall disease control rate at 80% [75].

IMM01 does not bind to human erythrocytes, avoiding "antigenic sink." Furthermore, following modification of deglycosylation, the immunogenicity of IMM01 is mitigated. As a result, IMM01 has good PK profiles, better tissue permeability and bioavailability, and better safety profiles, as reflected by the absence of anti-drug antibody (ADA) as well as no drug-related severe adverse events (SAEs) in the recruited subjects thus far.

Additionally, IMM01 showed strong synergistic anti-tumor activity when used in combination with other targeted drugs and immune checkpoint inhibitors in preclinical in vivo efficacy studies. Clinical applications of IMM01 in combination with azacitidine in the treatment of AML and MDS, with rituximab in the treatment of R/R CD20-positive B-cell lymphoma and with imatinib in the treatment of HER2-positive solid tumors in phase Ib/II stage have been approved by the NMPA to enter into clinical investigation [75].

IMM0306 is a bi-specific antibody trap (mAb-Trap) simultaneously targeting CD47 and CD20. IMM0306 was designed to bind CD20 with higher affinity than to bind CD47, thus avoiding the binding with CD47 in normal tissues so as to reduce the toxicity related to CD47 target. Preclinical in vivo efficacy studies indicated strong and robust anti-tumor activities leading to complete elimination of established tumor, even at the dose as low as 1.5 mg/kg (Unpublished data). At present, there is one ongoing clinical trial in the NCT system (NCT04746131) and another 1 in the CDT system (CTR20192612).

IMM2902 is another bi-specific antibody trap that simultaneously targets CD47 and Her2. This program has recently been approved both by NMPA and by FDA for phase I clinical trial for Her2-positive solid tumors. Preclinical studies revealed strong and robust anti-tumor activities against a variety of breast and gastric tumor models including Her2-low and trastuzumab-resistant breast tumors (Unpublished data, personal communications). The proposed mechanisms include improved ADCP, antibody-dependent cell-mediated cytotoxicity (ADCC) and accelerated Her2 degradation when the two targets were cross-linked by IMM2902.

### AK117

AK117 is an anti-CD47 mAb with a unique structure. It not only retains the anti-tumor activity, but also eliminates the agglutination of RBCs and significantly reduces the phagocytic activity of macrophages toward RBCs. As of August 30, 2021, there were 4 clinical trials ongoing in the NCT platform (NCT04900350 for MDS, NCT04980885 for AML, NCT04728334 and NCT04349969 for malignant neoplasms, respectively) (Table 1). Meanwhile, there were 3 ongoing clinical trials in the CDT system (CTR20211305, CTR20210825, CTR20202684) (Table 2).

A phase I clinical trial (NCT04349969) preliminary safety and receptor occupancy data from an ongoing dose-escalation study in 15 patients with advanced or metastatic solid tumors showed AK117 is safe and well tolerated with no infusion-related reactions (IRRs) or severe treatment-related adverse events (TRAEs) observed. AK117 does not require a lower “priming” dose to prevent anemia [76].

### SHR-1603

SHR-1603 is a humanized IgG4 mAb targeting CD47. Two validated liquid chromatography-mass spectrometry methods had been developed with different pretreatments for the quantification of an anti-CD47 mAb in rat and cynomolgus monkey serum compared with an electrochemiluminescence method. The established LC–MS methods exhibited superior accuracy, efficiency and cost-effectiveness for the PK assessment of SHR-1603 in the preclinical study [77]. At present, there is one clinical trial in the NCT (NCT03722186) and CDT (CTR20181964) systems in the suspended status.

### ZL-1201

ZL-1201 is an anti-human CD47 mAb. In July 2020, ZL-1201 injection targeting CD47 was approved for clinical research in China with patients of advanced solid tumor or malignant hematological tumor. Previously, it had been approved by FDA for clinical trial in USA. At present, there is one ongoing phase I clinical trial (NCT04257617) for ZL-1201 in NCT system for patients with advanced cancer and another one (CTR20210973) in the CDT system for patients with locally advanced solid tumor or hematological malignancy.

### JMT601 (CPO107)

JMT601 (CPO107) is a bi-specific SIRPα fusion protein with synergistic targeted binding effect on CD20 and CD47. It is based on the approved anti-CD20 antibody ofatumumab by adding the CD47 binding fragment SIRPα. It effectively binds to CD20 on the surface of tumor cells to induce antibody-dependent cell-mediated cytotoxicity (ADCC) and complement-dependent cytotoxicity (CDC). It combines with CD47 expressed on tumor cells to send a "do not eat me" signal to macrophages. JMT601 (CPO107) blocked the expression of CD47 on tumor cells and SIRPα on macrophages. Through interfering CD47/SIRPα, the interaction of JMT601 (CPO107) enhanced ADCP. Compared with the traditional CD20 antibody, in a number of human B-cell lymphoma models the efficacy of JMT601 (CPO107) was more obvious. The preclinical toxicological studies have shown that JMT601 (CPO107) had no significant binding to CD20 negative cells, and there was no significant involvement of CD47 strong positive cells such as RBCs and platelets. A phase I/II multicenter clinical trial (NCT04853329) had been approved in CD20 + NHL patients [78].

### Other potential candidates

There are several potential candidates underway for clinical research, such as LQ001, PT217, HLX-24. In short, the CD47-SIRPα signaling pathway has a great therapeutic potential, and CD47 has become another highly competitive target after PD-1/PD-L1 in cancer immunotherapy. In recent years, a large number of research activities have been conducted around the world in the research and development of CD47-targeted drugs.

## CD47 combination therapies

Immune checkpoint inhibitor (ICI) therapy, such as PD-1/PD-L1, is considered as a revolutionary anti-tumor immunotherapy strategy. However, certain tumors do not respond to the ICI therapy, owing to the lack of T lymphocytes pre-infiltration and co-existing of intricate immune-negative microenvironment including the macrophage-suppressed “Don’t eat me” CD47 signal overexpression. Therefore, studies to boost both the innate and adaptive immune reactions by cutting off PD-1/PD-L1 and CD47-SIRPa signal pathways have been tried. The preclinical experimental results showed that the nanocomposites exhibited a stronger anti-tumor immune effect, promoted infiltration of T lymphocyte into the tumor site and strengthened phagocytosis of macrophages. More importantly, it can significantly reverse pro-tumor M2-like tumor-associated macrophages (TAMs) to anti-tumor M1-like TAMs [79]. This provided a promising strategy to develop high-efficiency and low-toxicity immunotherapy based on nanotechnology.

BsAbs against dual checkpoint blocking of CD47 and PD-L1 have been developed to provide new insights into how bone marrow cells and T cells are uniquely regulated by dual congenital and adaptive checkpoint mAbs. Results demonstrated their potential in clinical development (NCT04881045) to improve patient prognosis through concurrent PD-L1 and CD47-targeted therapy [80]. Other BsAbs have also been developed by targeting different immune check point pathways (Table 1). Multiple clinical trials targeting dual molecules such as CD47/PD-1(HX009), CD47/41BB (DSP107), CD47/PD-L1 (IBI322, PF-07257876), CD47/CD20 (IMM0306), CD47/CD19 (TG-1801) have been started.

Based on the high expression of cytotoxic T lymphocyte-associated protein 4 (CTLA-4) on regulatory T (Treg) cells and abundant Fc receptor-expressing active phagocytes inside the TME, preclinical study had been done by combining anti-CTLA-4 mAbs with the SIRPα, targeting Treg cells and selectively blocking CD47 on intratumoral Treg cells, respectively. The results demonstrated that simultaneously modulating both "eat me" and “do not eat me” signals induced Treg cell depletion inside the TME and could be an effective strategy for treating solid tumors [81].

## Challenges for CD47 clinical development

CD47 is a target expressed in many cancers. Due to ubiquitous CD47 expression on senescent RBCs, however, the therapeutic utility of CD47-SIRPα blockade mAbs is largely compromised due to significant RBCs toxicities and fast target-mediated clearance as a result of extensive expression of CD47 on normal cells. A priming strategy has been developed to avoid the RBCs depletion toxicities. Patients are first treated at a low dose to remove the aging RBCs, thereby inducing compensatory hematopoiesis [10].

To overcome these limitations and further improve therapeutic efficacy, new generation CD47 antibodies that efficiently target tumor cells while exerting a minimal adverse effect on RBCs to avoid severe anemia were discovered [51]. A few ideally designed BsAbs have been developed, such as IBI322 for CD47/PD-L1, IMM0306 for CD47/D20 and IMM2902 for CD47/Her2, which can preferentially bind to CD47 on tumor cells with the help of higher affinity of the bi-specific molecules for another tumor target, effectively inhibiting CD47-SIRPα signal and triggering strong tumor cell phagocytosis in vitro, but only with minimal impact on CD47 single positive cells such as human RBCs. However, as discussed previously, multiple factors have to be considered such as target selection for signaling pathway, different subclasses with different effector functions such as ADCC and ADCP, antigen cellular distribution and the related biological activity [82].

The on-target/off-tumor effects, especially on platelets, can also cause potential problems with thrombocytopenia. However, this toxicity of anti-CD47 antibody on erythrocytes and platelets seems to be Fc dependent [51]. These findings suggest that optimizing the Fc structure of anti-CD47 mAs may ameliorate these adverse effects. Some of the new generation CD47 mAbs had been designed with this optimization. In addition, considering that old erythrocytes may be more susceptible to phagocytosis, the age of patients should be considered in the future CD47/SIRPα-

targeted therapy studies [83, 84].

The so-called antigen sink effect may also add challenges to the anti-CD47 therapy. The widespread expression of CD47 means that a drug may need a large initial dose and/or frequent administration to effectively block CD47. Compared with CD47, SIRPα has more limited histological distribution, which may have a better blocking effect and less therapeutic toxicities in targeted therapy [85]. Due to the polymorphisms of SIRP family members (SIRPβ and SIRPγ), cross-reactivity may occur between SIRP members. However, the consequences of targeting these different isoforms of receptors are not yet clear [86].

The anti-CD47 mAbs therapy may also face challenges in the combination therapies, including tumor cell-specific antibodies and T-cell checkpoint inhibitors. Usually a given anti-CD47 mAb is human IgG4 that has minimal activity of FcγR engagement and cannot stimulate the so-called Eat me signal on its own and thus would need another IgG1 antibody to fulfill the task. This will limit the use of CD47 mAb in combination with another IgG4 antibody (such as PD-1 antibody). Conversely, CD47-specific IgG1 antibody or recombinant protein would have the advantages of “One stone, two birds” if the hematological toxicity could be resolved. As mentioned earlier, IMM01 is such a molecule that can simultaneously block the “Don’t eat me” signal and stimulate the “Eat me” signal and thus would have little limitation when combining with other tumor therapeutics.

## Conclusions and future directions

From the laboratory to the bedside, different types of CD47-related drugs including mono-specific and BsAbs as well as fusion proteins have been developed into variable clinical research stages. Although IgG4 is the most popular isotype, however, other isotypes including IgG2 and IgG1 have been used with the consideration of multiple factors as it was discussed previously [82]. Promising results have been achieved from the clinical trials. New technologies for the development of CD47 mAbs have been explored in different therapeutic agents. In addition to CD47 blockades on target cells and SIRPα blockades on immune effector cells, the third category of CD47-SIRPα signaling pathway inhibitor is the glutaminyl-peptide cyclotransferase-like protein (QPCTL) which is required for CD47 maturation [87]. QPCTL could be a novel target to augment antibody therapy of cancer. Meanwhile, novel drug carriers such as modified biomimetic nanoparticles have been explored in cancer therapy [88], which may be a novel pathway worthy to deliver the therapeutic CD47 mAbs.

It is important to emphasize that because TME is so intricate that blocking single signaling pathways on a subset of immune cells only have limited or mild impact. For further investigation and better tumor treatment efficacy, multi-target combination therapies are particularly important. By precisely and comprehensively reprogramming TME, combination therapies containing CD47 and other immune checkpoint inhibitors may exert better effects than monotherapy. Considering the nature of SIRPα-expressing macrophages and dendritic cells as professional antigen-presenting cells [88, 89], concurrently activating both innate immune cells via CD47-SIRPα blockade and adaptive immune cells via immune checkpoint inhibitors in conjunction with other tumor-targeted therapies so to achieve long-lasting anti-tumor activity would be the future of drug development for tumor immunotherapy. Most recently, a study demonstrated that SIRPα-αCD123 antibodies combined local CD47 blockade with specific AML LSCs targeting in a single molecule, minimized the risk of targeting healthy cells and efficiently eliminated AML LSCs. This indicates that SIRPα-αCD123 fusion antibodies targeting CD123 in conjunction with CD47 blockade could enhance the clearance of AML-initiating cells, which may be used as promising therapeutic interventions for AML [90].

## Data Availability

All clinical trials-related information was obtained from public databases or from the related company’s announcement or website information, except the information from personal communications.

## Abbreviations

ADCC: Antibody-dependent cellular cytotoxicity
ADCP: Antibody-dependent cellular phagocytosis
AML: Acute myeloid leukemia
B-NHL: B-cell non-Hodgkin lymphoma
BsAbs: Bi-specific antibody
CD47: Cluster of differentiation 47
CDC: Complement-dependent cytotoxicity
CR: Complete response
CTLA-4: Cytotoxic T lymphocyte-associated protein 4
CTR: China drug trials registry
DLBCL: Diffuse large B-cell lymphoma
FcgR: Fc gamma receptor
GC: Gastric cancer
HA: Hemagglutination
HNSC: Head and neck squamous cell carcinoma
ICI: Immune checkpoint inhibitor
IRRs: Infusion-related reactions
LMS: Leiomyosarcoma
LSC: Leukemia stem cells
mAbs: Monoclonal antibody
MDS: Myelodysplastic syndrome
MLFS: Morphologic leukemia-free state
MM: Multiple myeloma
MOA: Mechanism of action
NCT: National clinical trials registry
NHL: Non-Hodgkin's lymphoma
NMPA: National Medical Products Administration
non-GCB: On-germinal center B-cell
ORR: Objective response rate
OS: Overall survival
PD-1/PD-L1: Programmed death-1, programmed death ligand-1
PR: Partial response
R/R NHL: Recurrent or refractory non-Hodgkin's lymphoma
RBCs: Red blood cells
SD: Stable disease
SIRPα: Signal regulatory protein α
ST: Solid tumor
TAMs: Tumor-associated macrophages
TME: Tumor microenvironment
T-NHL: T-cell non-Hodgkin lymphoma
TRAEs: Treatment-related adverse events
Treg: Regulatory T
VEGF-A: Vascular endothelial growth factor-A
